# 10-Benzyl-10*H*-phenothia­zine 9-oxide

**DOI:** 10.1107/S160053680902577X

**Published:** 2009-07-08

**Authors:** Zhouqing Xu, Yanchun Sun, Lei Yang, Qiang Wang

**Affiliations:** a Department of Physics & Chemistry, Henan Polytechnic University, Jiao Zuo 454000, People’s Republic of China; b Department of Medicine, Hebi College of Vocation and Technology, He Bi 458030, People’s Republic of China

## Abstract

In the title compound, C_19_H_15_NOS, the butterfly angle between the mean planes defined by the S, N and phenyl C atoms of the two wings of the phenothiazine unit is 23.4 (1)°. In the crystal, a supra­molecular two-dimensional arrangement arises from weak inter­molecular C—H⋯O inter­actions.

## Related literature

For applications of phenothia­zines, see: Miller *et al.* (1999 ▶); Wermuth (2003 ▶); Wang *et al.* (2008 ▶); Lam *et al.* (2001 ▶). For the synthesis, see: Zhu *et al.* (2006 ▶); Gilman *et al.* (1954 ▶).
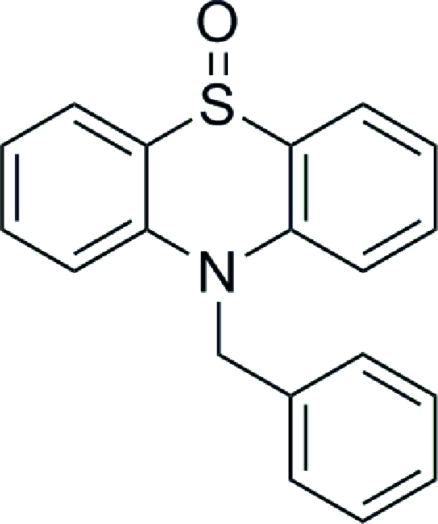


## Experimental

### 

#### Crystal data

C_19_H_15_NOS
*M* *_r_* = 305.38
Monoclinic, 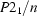

*a* = 6.2819 (4) Å
*b* = 11.9259 (8) Å
*c* = 20.3220 (14) Å
β = 94.6140 (10)°
*V* = 1517.54 (18) Å^3^
*Z* = 4
Mo *K*α radiation
μ = 0.21 mm^−1^
*T* = 296 K
0.30 × 0.22 × 0.19 mm

#### Data collection

Bruker APEXII CCD area-detector diffractometer
Absorption correction: multi-scan (*SADABS*; Sheldrick, 2003 ▶) *T*_min_ = 0.939, *T*_max_ = 0.961
7251 measured reflections
2511 independent reflections
1872 reflections with *I* > 2σ(*I*)
*R*_int_ = 0.032

#### Refinement

*R*[*F* ^2^ > 2σ(*F* ^2^)] = 0.036
*wR*(*F* ^2^) = 0.094
*S* = 1.02
2511 reflections
199 parameters
H-atom parameters constrained
Δρ_max_ = 0.17 e Å^−3^
Δρ_min_ = −0.23 e Å^−3^

### 

Data collection: *APEX2* (Bruker, 2003 ▶); cell refinement: *SAINT* (Bruker, 2001 ▶); data reduction: *SAINT*; program(s) used to solve structure: *SHELXS97* (Sheldrick, 2008 ▶); program(s) used to refine structure: *SHELXL97* (Sheldrick, 2008 ▶); molecular graphics: *SHELXTL* (Sheldrick, 2008 ▶); software used to prepare material for publication: *SHELXTL*.

## Supplementary Material

Crystal structure: contains datablocks I, global. DOI: 10.1107/S160053680902577X/pv2174sup1.cif

Structure factors: contains datablocks I. DOI: 10.1107/S160053680902577X/pv2174Isup2.hkl

Additional supplementary materials:  crystallographic information; 3D view; checkCIF report

## Figures and Tables

**Table 1:** Hydrogen-bond geometry (Å, °)

*D*—H⋯*A*	*D*—H	H⋯*A*	*D*⋯*A*	*D*—H⋯*A*
C13—H13*B*⋯O1^i^	0.97	2.52	3.431 (2)	157
C18—H18⋯O1^ii^	0.93	2.57	3.442 (3)	157

Symmetry codes: (i) 
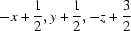
; (ii) 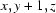.

**Fig. 1.**
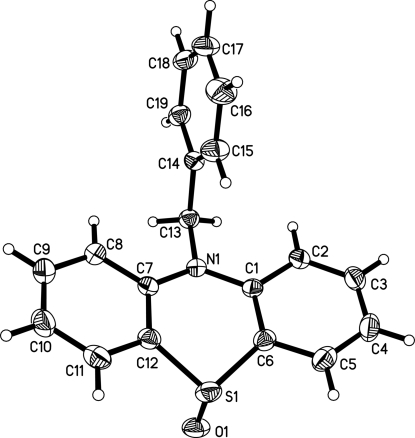
The molecular structure of the title molecule, with the atom-numbering scheme. Displacement ellipsoids are drawn at the 30% probability level.

**Fig. 2.**
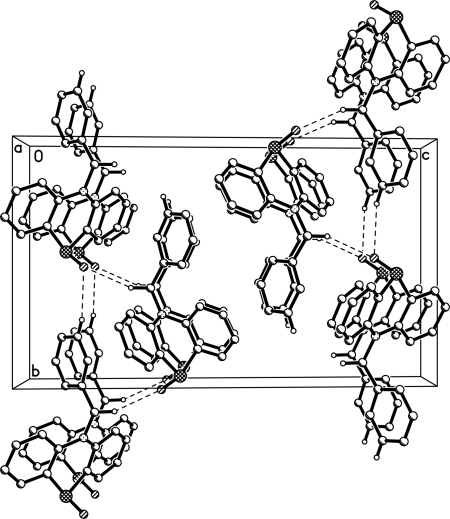
A partial packing diagram of the title compound. Hydrogen bonds are shown as dashed lines, viewed down the a axis.

## supplementary crystallographic information

### Comment

Phenothiazine molecule is a well known heterocycle. The phenothiazine structure occurs in many synthetic dyes, electroluminescent materials (Miller *et al.*,  1999) and drugs, especially various antipsychotic drugs, e.g., chlorpromazine and promethazine (Wermuth, 2003). Recently, some new applications of phenothazine derivatives have been found in medicines, such as antitubercular (Wang *et al.*,  2008) and antitumor (Lam *et al.*,  2001). As a part of our programme devoted to the new applications of phenothazine derivatives in medicne, we report herein the crystal structure of the title compound, (I).

The molecular structure of (I) is shown in Fig. 1, with its respective labels. The butterfly angle between the mean-planes defined by atoms S1/N1/C1-C6 and S1/N1/C7-C12 is 23.4 (1) °. The crystal packing (Fig. 2) consists of two-dimensional infinite plane along the *a* axis generated by intermolecular interactions of the weak C—H···O hydrogen bonds (details are in Table 1).

### Experimental

All reagents were of analytical grade. The title compound was prepared according to a literature method (Zhu *et al.*,  2006; Gilman *et al.*,  1954) from N-benzylphenothiazine. The purity of the compound was checked by determining its melting point. It was characterized by recording its infrared spectra and elemental analyses. Single crystals of the title compound were obtained by slow evaporation of its ethanol solution. The X-ray diffraction studies were made at room temperature.

### Refinement

All H atoms were included in calculated positions, with C—H bond lengths fixed at 0.97 Å (methylene CH_2_) and 0.93Å (aryl group) and were refined in the riding-model approximation. *U*_iso_(H) values were allowed at 1.2 times *U*_eq_(C).

### Crystal data

**Table:**

C_19_H_15_NOS	*F*(000) = 640
*M**_r_* = 305.38	*D*_x_ = 1.337 Mg m^−^^3^
Monoclinic, *P*2_1_/*n*	Mo *K*α radiation, λ = 0.71073 Å
Hall symbol: -P 2yn	Cell parameters from 1664 reflections
*a* = 6.2819 (4) Å	θ = 2.6–22.2°
*b* = 11.9259 (8) Å	µ = 0.21 mm^−^^1^
*c* = 20.3220 (14) Å	*T* = 296 K
β = 94.614 (1)°	Block, yellow
*V* = 1517.54 (18) Å^3^	0.30 × 0.22 × 0.19 mm
*Z* = 4	

### Data collection

**Table:**

Bruker SMART CCD area-detector diffractometer	2511 independent reflections
Radiation source: fine-focus sealed tube	1872 reflections with *I* > 2σ(*I*)
graphite	*R*_int_ = 0.032
φ and ω scans	θ_max_ = 24.5°, θ_min_ = 2.0°
Absorption correction: multi-scan (*SADABS*; Sheldrick, 2003)	*h* = −7→6
*T*_min_ = 0.939, *T*_max_ = 0.961	*k* = −13→13
7251 measured reflections	*l* = −23→23

### Refinement

**Table:**

Refinement on *F*^2^	Primary atom site location: structure-invariant direct methods
Least-squares matrix: full	Secondary atom site location: difference Fourier map
*R*[*F*^2^ > 2σ(*F*^2^)] = 0.036	Hydrogen site location: inferred from neighbouring sites
*wR*(*F*^2^) = 0.094	H-atom parameters constrained
*S* = 1.02	*w* = 1/[σ^2^(*F*_o_^2^) + (0.0425*P*)^2^ + 0.2518*P*] where *P* = (*F*_o_^2^ + 2*F*_c_^2^)/3
2511 reflections	(Δ/σ)_max_ = 0.001
199 parameters	Δρ_max_ = 0.17 e Å^−^^3^
0 restraints	Δρ_min_ = −0.23 e Å^−^^3^

### Special details

**Table:**

Geometry. All esds (except the esd in the dihedral angle between two l.s. planes) are estimated using the full covariance matrix. The cell esds are taken into account individually in the estimation of esds in distances, angles and torsion angles; correlations between esds in cell parameters are only used when they are defined by crystal symmetry. An approximate (isotropic) treatment of cell esds is used for estimating esds involving l.s. planes.
Refinement. Refinement of F^2^ against ALL reflections. The weighted R-factor wR and goodness of fit S are based on F^2^, conventional R-factors R are based on F, with F set to zero for negative F^2^. The threshold expression of F^2^ > 2sigma(F^2^) is used only for calculating R-factors(gt) etc. and is not relevant to the choice of reflections for refinement. R-factors based on F^2^ are statistically about twice as large as those based on F, and R- factors based on ALL data will be even larger.

### Fractional atomic coordinates and isotropic or equivalent isotropic displacement parameters (Å^2^)

**Table:**

	*x*	*y*	*z*	*U*_iso_*/*U*_eq_	
N1	0.2291 (2)	0.28879 (12)	0.65659 (8)	0.0377 (4)	
O1	0.2735 (3)	−0.01747 (11)	0.65821 (7)	0.0601 (4)	
S1	0.11841 (9)	0.04344 (4)	0.61145 (3)	0.04963 (19)	
C1	0.0471 (3)	0.24089 (15)	0.67982 (9)	0.0379 (5)	
C2	−0.0702 (3)	0.29706 (17)	0.72559 (10)	0.0470 (5)	
H2	−0.0251	0.3672	0.7411	0.056*	
C3	−0.2502 (3)	0.2501 (2)	0.74777 (12)	0.0559 (6)	
H3	−0.3216	0.2879	0.7794	0.067*	
C4	−0.3281 (4)	0.1485 (2)	0.72440 (12)	0.0615 (7)	
H4	−0.4534	0.1190	0.7387	0.074*	
C5	−0.2173 (3)	0.09217 (19)	0.67984 (11)	0.0553 (6)	
H5	−0.2685	0.0235	0.6636	0.066*	
C6	−0.0284 (3)	0.13530 (16)	0.65792 (10)	0.0429 (5)	
C7	0.3155 (3)	0.25083 (15)	0.59956 (9)	0.0385 (5)	
C8	0.4588 (3)	0.31695 (18)	0.56709 (11)	0.0490 (5)	
H8	0.4994	0.3865	0.5845	0.059*	
C9	0.5405 (4)	0.2812 (2)	0.51022 (12)	0.0613 (6)	
H9	0.6383	0.3261	0.4904	0.074*	
C10	0.4801 (4)	0.1798 (2)	0.48169 (12)	0.0644 (7)	
H10	0.5326	0.1571	0.4423	0.077*	
C11	0.3413 (4)	0.11342 (18)	0.51272 (11)	0.0551 (6)	
H11	0.3003	0.0447	0.4942	0.066*	
C12	0.2605 (3)	0.14667 (16)	0.57131 (10)	0.0424 (5)	
C13	0.3051 (3)	0.39597 (15)	0.68456 (10)	0.0400 (5)	
H13A	0.4581	0.4007	0.6815	0.048*	
H13B	0.2798	0.3974	0.7310	0.048*	
C14	0.2012 (3)	0.49772 (15)	0.65152 (9)	0.0380 (5)	
C15	0.0249 (4)	0.49180 (19)	0.60717 (11)	0.0571 (6)	
H15	−0.0329	0.4223	0.5951	0.068*	
C16	−0.0678 (4)	0.5888 (2)	0.58025 (13)	0.0713 (7)	
H16	−0.1875	0.5843	0.5503	0.086*	
C17	0.0174 (4)	0.6915 (2)	0.59780 (13)	0.0658 (7)	
H17	−0.0449	0.7566	0.5799	0.079*	
C18	0.1927 (4)	0.69818 (18)	0.64129 (12)	0.0577 (6)	
H18	0.2498	0.7679	0.6531	0.069*	
C19	0.2860 (3)	0.60252 (16)	0.66792 (10)	0.0470 (5)	
H19	0.4072	0.6080	0.6972	0.056*	

### Atomic displacement parameters (Å^2^)

**Table:**

	*U*^11^	*U*^22^	*U*^33^	*U*^12^	*U*^13^	*U*^23^
N1	0.0403 (9)	0.0307 (9)	0.0428 (9)	0.0004 (8)	0.0072 (7)	0.0001 (7)
O1	0.0760 (11)	0.0362 (8)	0.0685 (10)	0.0096 (8)	0.0085 (9)	0.0108 (7)
S1	0.0607 (4)	0.0344 (3)	0.0537 (4)	−0.0069 (3)	0.0040 (3)	−0.0047 (2)
C1	0.0382 (11)	0.0337 (10)	0.0415 (11)	0.0043 (9)	0.0021 (9)	0.0082 (9)
C2	0.0483 (13)	0.0402 (12)	0.0538 (13)	0.0088 (10)	0.0121 (10)	0.0072 (10)
C3	0.0492 (14)	0.0589 (15)	0.0617 (15)	0.0157 (12)	0.0167 (11)	0.0140 (12)
C4	0.0402 (13)	0.0721 (17)	0.0739 (17)	0.0005 (13)	0.0153 (12)	0.0190 (14)
C5	0.0473 (13)	0.0522 (13)	0.0655 (15)	−0.0094 (11)	−0.0006 (12)	0.0110 (12)
C6	0.0419 (12)	0.0407 (12)	0.0458 (12)	−0.0003 (10)	0.0012 (9)	0.0053 (9)
C7	0.0375 (11)	0.0346 (11)	0.0437 (12)	0.0061 (9)	0.0047 (9)	0.0040 (9)
C8	0.0474 (13)	0.0426 (12)	0.0586 (14)	0.0012 (10)	0.0142 (11)	0.0047 (11)
C9	0.0613 (15)	0.0594 (15)	0.0664 (16)	0.0094 (13)	0.0250 (12)	0.0106 (13)
C10	0.0782 (18)	0.0658 (17)	0.0527 (15)	0.0166 (15)	0.0258 (13)	0.0029 (13)
C11	0.0688 (16)	0.0474 (13)	0.0493 (13)	0.0133 (12)	0.0061 (12)	−0.0056 (11)
C12	0.0449 (12)	0.0386 (11)	0.0435 (12)	0.0064 (10)	0.0032 (9)	0.0025 (9)
C13	0.0406 (11)	0.0351 (11)	0.0441 (11)	−0.0019 (9)	0.0024 (9)	−0.0017 (9)
C14	0.0405 (12)	0.0341 (11)	0.0398 (11)	0.0028 (9)	0.0059 (9)	−0.0015 (9)
C15	0.0581 (15)	0.0467 (13)	0.0640 (15)	−0.0007 (11)	−0.0105 (12)	0.0036 (11)
C16	0.0649 (17)	0.0728 (18)	0.0735 (17)	0.0149 (15)	−0.0109 (13)	0.0165 (15)
C17	0.0839 (19)	0.0470 (15)	0.0682 (16)	0.0211 (14)	0.0174 (15)	0.0187 (12)
C18	0.0831 (18)	0.0347 (12)	0.0580 (14)	0.0000 (12)	0.0225 (13)	0.0035 (11)
C19	0.0572 (14)	0.0402 (12)	0.0443 (12)	−0.0051 (11)	0.0078 (10)	−0.0017 (10)

### Geometric parameters (Å, °)

**Table:**

N1—C1	1.394 (2)	C9—C10	1.381 (3)
N1—C7	1.394 (2)	C9—H9	0.9300
N1—C13	1.463 (2)	C10—C11	1.368 (3)
O1—S1	1.4934 (15)	C10—H10	0.9300
S1—C6	1.756 (2)	C11—C12	1.389 (3)
S1—C12	1.759 (2)	C11—H11	0.9300
C1—C2	1.402 (3)	C13—C14	1.509 (2)
C1—C6	1.405 (3)	C13—H13A	0.9700
C2—C3	1.370 (3)	C13—H13B	0.9700
C2—H2	0.9300	C14—C15	1.372 (3)
C3—C4	1.377 (3)	C14—C19	1.389 (3)
C3—H3	0.9300	C15—C16	1.388 (3)
C4—C5	1.363 (3)	C15—H15	0.9300
C4—H4	0.9300	C16—C17	1.372 (3)
C5—C6	1.399 (3)	C16—H16	0.9300
C5—H5	0.9300	C17—C18	1.358 (3)
C7—C12	1.400 (3)	C17—H17	0.9300
C7—C8	1.401 (3)	C18—C19	1.374 (3)
C8—C9	1.370 (3)	C18—H18	0.9300
C8—H8	0.9300	C19—H19	0.9300
			
C1—N1—C7	122.21 (16)	C11—C10—C9	118.5 (2)
C1—N1—C13	118.50 (15)	C11—C10—H10	120.8
C7—N1—C13	118.04 (15)	C9—C10—H10	120.8
O1—S1—C6	107.77 (9)	C10—C11—C12	121.3 (2)
O1—S1—C12	107.82 (9)	C10—C11—H11	119.3
C6—S1—C12	96.94 (9)	C12—C11—H11	119.3
N1—C1—C2	121.18 (17)	C11—C12—C7	120.65 (19)
N1—C1—C6	121.68 (18)	C11—C12—S1	115.57 (16)
C2—C1—C6	117.14 (18)	C7—C12—S1	123.25 (15)
C3—C2—C1	121.0 (2)	N1—C13—C14	114.44 (15)
C3—C2—H2	119.5	N1—C13—H13A	108.6
C1—C2—H2	119.5	C14—C13—H13A	108.6
C2—C3—C4	121.7 (2)	N1—C13—H13B	108.6
C2—C3—H3	119.1	C14—C13—H13B	108.6
C4—C3—H3	119.1	H13A—C13—H13B	107.6
C5—C4—C3	118.5 (2)	C15—C14—C19	118.54 (18)
C5—C4—H4	120.8	C15—C14—C13	123.23 (17)
C3—C4—H4	120.8	C19—C14—C13	118.22 (17)
C4—C5—C6	121.5 (2)	C14—C15—C16	120.5 (2)
C4—C5—H5	119.3	C14—C15—H15	119.8
C6—C5—H5	119.3	C16—C15—H15	119.8
C5—C6—C1	120.1 (2)	C17—C16—C15	119.9 (2)
C5—C6—S1	115.95 (16)	C17—C16—H16	120.0
C1—C6—S1	123.43 (15)	C15—C16—H16	120.0
N1—C7—C12	121.97 (17)	C18—C17—C16	120.0 (2)
N1—C7—C8	121.10 (18)	C18—C17—H17	120.0
C12—C7—C8	116.93 (19)	C16—C17—H17	120.0
C9—C8—C7	121.4 (2)	C17—C18—C19	120.4 (2)
C9—C8—H8	119.3	C17—C18—H18	119.8
C7—C8—H8	119.3	C19—C18—H18	119.8
C8—C9—C10	121.2 (2)	C18—C19—C14	120.6 (2)
C8—C9—H9	119.4	C18—C19—H19	119.7
C10—C9—H9	119.4	C14—C19—H19	119.7
			
C7—N1—C1—C2	−162.60 (17)	C7—C8—C9—C10	1.7 (3)
C13—N1—C1—C2	4.4 (3)	C8—C9—C10—C11	−2.1 (4)
C7—N1—C1—C6	16.6 (3)	C9—C10—C11—C12	0.5 (3)
C13—N1—C1—C6	−176.46 (16)	C10—C11—C12—C7	1.5 (3)
N1—C1—C2—C3	179.35 (18)	C10—C11—C12—S1	−170.45 (17)
C6—C1—C2—C3	0.1 (3)	N1—C7—C12—C11	176.90 (18)
C1—C2—C3—C4	−2.6 (3)	C8—C7—C12—C11	−1.8 (3)
C2—C3—C4—C5	2.4 (3)	N1—C7—C12—S1	−11.8 (3)
C3—C4—C5—C6	0.1 (3)	C8—C7—C12—S1	169.48 (15)
C4—C5—C6—C1	−2.5 (3)	O1—S1—C12—C11	90.65 (17)
C4—C5—C6—S1	169.87 (17)	C6—S1—C12—C11	−158.12 (16)
N1—C1—C6—C5	−176.90 (17)	O1—S1—C12—C7	−81.06 (18)
C2—C1—C6—C5	2.3 (3)	C6—S1—C12—C7	30.17 (18)
N1—C1—C6—S1	11.3 (3)	C1—N1—C13—C14	−86.1 (2)
C2—C1—C6—S1	−169.46 (15)	C7—N1—C13—C14	81.4 (2)
O1—S1—C6—C5	−90.78 (17)	N1—C13—C14—C15	11.1 (3)
C12—S1—C6—C5	157.95 (16)	N1—C13—C14—C19	−170.27 (17)
O1—S1—C6—C1	81.32 (17)	C19—C14—C15—C16	−1.0 (3)
C12—S1—C6—C1	−29.95 (18)	C13—C14—C15—C16	177.7 (2)
C1—N1—C7—C12	−16.3 (3)	C14—C15—C16—C17	0.2 (4)
C13—N1—C7—C12	176.65 (16)	C15—C16—C17—C18	0.2 (4)
C1—N1—C7—C8	162.34 (17)	C16—C17—C18—C19	0.1 (4)
C13—N1—C7—C8	−4.7 (3)	C17—C18—C19—C14	−0.9 (3)
N1—C7—C8—C9	−178.49 (19)	C15—C14—C19—C18	1.3 (3)
C12—C7—C8—C9	0.2 (3)	C13—C14—C19—C18	−177.42 (19)

### Hydrogen-bond geometry (Å, °)

**Table:**

*D*—H···*A*	*D*—H	H···*A*	*D*···*A*	*D*—H···*A*
C13—H13B···O1^i^	0.97	2.52	3.431 (2)	157
C18—H18···O1^ii^	0.93	2.57	3.442 (3)	157

Symmetry codes: (i) −*x*+1/2, *y*+1/2, −*z*+3/2; (ii) *x*, *y*+1, *z*.
