# 10-Benzyl-10*H*-phenothia­zine 9-oxide. Corrigendum

**DOI:** 10.1107/S1600536810001996

**Published:** 2010-01-30

**Authors:** Zhouqing Xu, Yanchun Sun, Lei Yang, Qiang Wang

**Affiliations:** a Department of Physics & Chemistry, Henan Polytechnic University, Jiao Zuo 454000, People’s Republic of China; b Department of Medicine, Hebi College of Vocation and Technology, He Bi 458030, People’s Republic of China

## Abstract

The chemical name of the title compound in the paper by Xu, Sun, Yang & Wang [*Acta Cryst.* (2009), E**65**, o1799] is corrected.

## Abstract 2

Corrigendum to *Acta Cryst.* (2009), E**65**, o1799.

In the paper by Xu *et al.* (2009) ▶, the chemical name given in the *Title* should be ‘10-Benzyl-10*H*-phenothia­zine 5-oxide’.
